# Simulation of Z-Shaped Graphene Geometric Diodes Using Particle-in-Cell Monte Carlo Method in the Quasi-Ballistic Regime

**DOI:** 10.3390/nano11092361

**Published:** 2021-09-11

**Authors:** John Stearns, Garret Moddel

**Affiliations:** Department of Electrical, Computer, and Energy Engineering, University of Colorado, Boulder, CO 80309-0425, USA; john.stearns@colorado.edu

**Keywords:** diode, rectenna, graphene, ballistic transport, quasi-ballistic transport, Monte Carlo simulation, particle-in-cell, simulation

## Abstract

Geometric diodes are planar conductors patterned asymmetrically to provide electrical asymmetry, and they have exhibited high-frequency rectification in infrared rectennas. These devices function by ballistic or quasi-ballistic transport in which the transport characteristics are sensitive to the device geometry. Common methods for predicting device performance rely on the assumption of totally ballistic transport and neglect the effects of electron momentum relaxation. We present a particle-in-cell Monte Carlo simulation method that allows the prediction of the current–voltage characteristics of geometric diodes operating quasi-ballistically, with the mean-free-path length shorter than the critical device dimensions. With this simulation method, we analyze a new diode geometry made from graphene that shows an improvement in rectification capability over previous geometries. We find that the current rectification capability of a given geometry is optimized for a specific mean-free-path length, such that arbitrarily large mean-free-path lengths are not desirable. These results present a new avenue for understanding geometric effects in the quasi-ballistic regime and show that the relationship between device dimensions and the carrier mean-free-path length can be adjusted to optimize device performance.

## 1. Introduction

We defined geometric diodes as planar conductors patterned with a geometric asymmetry that gives rise to a preferred current direction in 2008 [1], and we have demonstrated that they rectify at DC and infrared frequencies [2,3]. Since these diodes are planar and therefore have extremely low capacitance, they have the potential to provide ultra-fast rectification. They can be used in rectennas, diodes coupled to antennas, to rectify infrared signals and produce DC power [3], and they can also be used in other applications that demand high-speed electronics. Other high-frequency diodes, such as metal–insulator–insulator–metal diodes, have substantially higher capacitance due to their parallel-plate configuration and hence lower operating frequencies [4] even for ideal materials [5]. In addition, geometric diodes may be able to achieve higher current asymmetries than other high-frequency diodes [6].

The mean-free-path length (*λ_MFP_*) of charge carriers in the geometric diode material is critical. For a geometric diode to exhibit rectification, charge carriers must travel ballistically (*λ_MFP_* greater than device dimensions) or quasi-ballistically (*λ_MFP_* near device dimensions) through the patterned material, which requires that device dimensions be on the order of *λ_MFP_* or lower. This imposes difficult fabrication requirements for devices made of conventional metals, as typical mean-free-path lengths are below 100 nm. This difficulty can be relaxed somewhat by using more exotic materials, such as graphene, which has shown mean-free-path lengths on the micron scale [7]. Such graphene devices have been demonstrated experimentally [2,3,8] and are capable of infrared rectification.

Methods of modeling geometric diodes often incorporate the Landauer–Buttiker formalism to obtain device current–voltage (*I(V)*) characteristics, using a transmission function obtained theoretically [9] or through simulation [10,11]. This approach is elegant but so far has been applied only to purely ballistic transport and does not include the momentum relaxation that is present in quasi-ballistic devices, which can still exhibit rectifying behavior. Another simulation method incorporates a Monte Carlo algorithm with Drude-like carrier transport through the devices, and it includes momentum relaxation by way of scattering events [3,12]. In this approach, the device geometry is established, and the input voltage is applied across the device at the two electrodes in order to compute the geometry-dependent electric field, which is assumed to be constant throughout the simulation time. Then, a certain number of charge carriers, each of which represents multiple electrons, are generated at random locations in the device, each with a randomly directed velocity with magnitude equal to the Fermi velocity (*v_F_*). Then, the carriers are allowed to move under the influence of the electric field over a small timestep, with the electric field perturbing their initial randomly directed momentum. If a charge carrier collides with a device boundary, it undergoes specular reflection and is allowed to continue propagating. If it crosses either of the electrodes, then a counter is incremented before another carrier is injected from the opposite electrode. Once a certain carrier has traveled a distance equal to the mean-free-path length, it is given a new randomly directed velocity, with magnitude *v_F_*, and the process repeats for a predetermined number of time steps. After a chosen time has elapsed, the current is derived from the counter and the total simulation time.

We present a new simulation method that incorporates a particle-in-cell algorithm (PIC) [13] to determine the *I(V)* characteristic for a given device. This method, which builds on the previous Monte Carlo method [3], decreases the computation time and includes a self-consistent electric field computation at every time step. Our application of this method also includes a more accurate implementation of the collision time statistics from the Drude model. Since this algorithm includes momentum relaxation, it is useful for the simulation of both ballistic and quasi-ballistic devices. Our previous simulations and demonstrations made use of an inverse-arrowhead design [3]. Here, we use this simulator to demonstrate a new diode geometry, the Z-diode, which shows improved current asymmetry over previous designs.

## 2. Materials and Methods

The simulation begins by establishing the diode boundaries in MATLAB [14] and creating a triangular mesh within them. The nodes of the mesh are the discrete points at which the charge concentration, electric potential, and electric field will be computed later in the algorithm. Then, a certain number of macroparticles, each of which can represent multiple electrons, are scattered within the device at random locations. The use of macroparticles, where each one typically represents 100 or 200 individual electrons depending on the carrier concentration of the material, facilitates quicker computation than simulating individual carriers. Each macroparticle is initially distributed to the three mesh nodes surrounding it using a weighting determined from the relative distances between the particle and each of the nodes. This establishes the initial spatial charge density. There is assumed to be a homogenous distribution of background positive charge that maintains charge neutrality in the device as a whole. Each of the macroparticles is initiated with a randomly directed velocity vector with magnitude equal to *v_F_*. The probabilistic nature of electron collisions is incorporated by assigning each macroparticle a time interval in the future at which it will undergo a momentum relaxation event. These times are sampled from an exponential decay distribution where *e^−t/^^τ^* represents the probability that an electron picked at random will have no collision during the next time interval *t*, and *τ* *=* *λ_MFP_/v_F_* represents the average momentum relaxation time of the choice material [15].

**Figure 1:**
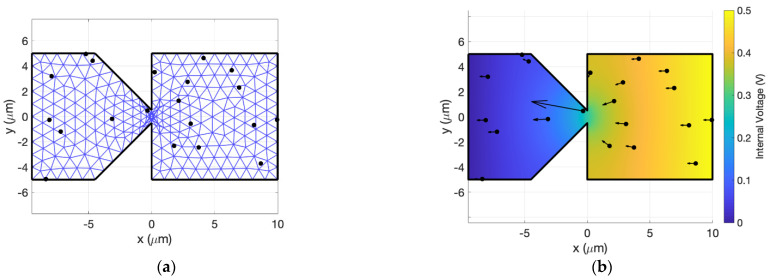
Particle-in-cell setup for an inverse-arrowhead diode with 20 macroparticles. (**a**) The particles are first scattered to the mesh defined within the diode geometry. (**b**) Then, the simulator uses a PDE solver to determine the voltage everywhere inside the diode given the input applied voltage, 0.5 V in this example. The arrows represent the electric field acting on each macroparticle.

The input voltage is set as a boundary condition between the two electrodes. MATLAB’s PDE package is used to solve Poisson’s equation for the potential at every mesh node. Then, the electric field is computed from the gradient of the solution. Then, the field at each particle is determined from the field at the surrounding three nodes using the distance weighting that was used for the charge distribution. The field exerts a force on the particle, changing its velocity over a small time-step *dt*, and the particles hop forward in time along the new velocity vector. The counter that tracks the time at which a given particle will undergo a momentum-destroying collision is decremented by *dt* for each particle. The new locations of the particles are used to compute the charge density again, and the process repeats itself many times. This methodology, which is usually incorporated in PIC algorithms, computes inter-particle interactions through the spatial charge density rather than through Coulombic forces between particle pairs, which would be more computationally expensive. Figure 1 shows the setup and results graphically for an inverse-arrowhead diode shape [3]. Note that for clarity, the figure contains only 20 macroparticles, but in full simulations, hundreds are used. For the simulations described herein, we always ensured that a sufficient number of macroparticles were used so that the results did not change with a further increase in the number of macroparticles. For a lower number of macroparticles, each macroparticle represents a larger number of individual electrons. Since inter-electron forces are not considered among electrons within the same macroparticle, more electron interactions are ignored and care must be given to ensure error is not introduced.

At any step, if the time-to-next-collision counter goes below zero for any particle, that particle’s momentum is reset by giving it a new randomly-directed velocity with magnitude *v_F_*, and its counter is reset from a sampling of the exponential decay distribution. The new counter value is reduced by the amount of time the particle traveled after the collision event, i.e., the previous negative value of the counter. This ensures that the momentum relaxation statistics are obeyed throughout the simulation and, on average, the particles will travel approximately a distance of *λ_MFP_* between collisions. If a particle collides with a device boundary, it is reflected specularly and continues propagating. When a particle crosses one of the electrode boundaries, a counter, *Q*, which counts the total number of charges to pass through the device throughout the entire simulation, is incremented or decremented accordingly. Then, the total current, *I*, is computed as,

(1) $$I=\frac{nA}{N_{macro}}\frac{qQ}{N_{t}dt}$$

where *n* is the material’s free carrier concentration per unit area, *A* is the total device area, *N_macro_* is the number of macroparticles used in the simulation, *q* is the elementary charge, and *N_t_* is the total number of simulated time steps.

It is important to note that *dt* must be chosen carefully with regard to *τ*. Since a particle’s collision counter is decremented by *dt* at every timestep, for larger *dt*, there is a larger margin by which a particle’s collision counter can drop below zero. This corresponds to the particle overshooting its actual collision location, which results in an unintentional increase in the distance traveled between collisions. Then, the simulated current will be overstated due to an artifactual increase in mobility. To avoid this problem, mesh elements must be small enough to capture the large potential gradients that occur at geometric constrictions in the device (see the large electric field acting on the particle near the constriction at *x = 0* in Figure 1b). If the mesh is too coarse, large potential gradients (electric fields) will be understated and yield a lower simulated current. On the other hand, arbitrarily small timesteps and mesh sizes require long simulation times. For each geometry, we repeated simulations for decreasing *dt* and mesh size to find values for each where the resulting *I(V)* remained approximately constant with further reductions. Once we determined a sufficiently small timestep and mesh size to give a grid-independent solution for a given geometry, we used them for all subsequent simulations of that geometry. A time step of dt=τ/10 was used for all simulations herein. For devices with geometric constrictions, the mesh element size varied throughout with a larger mesh size near the electrodes and a smaller mesh size near the constrictions.

## 3. Results

### 3.1. Rectangular Strip

**Figure 2:**
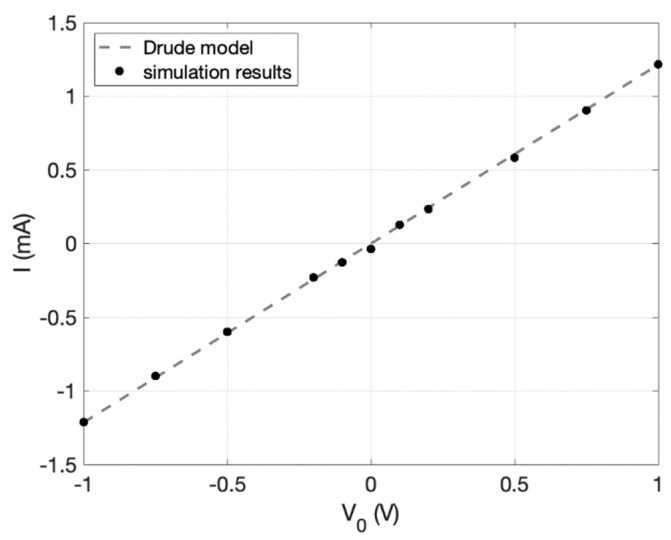
Verification of the simulation algorithm’s current–voltage prediction. The simulator was run for a simple rectangular strip of length 10 μm and width 2.5 μm. The carrier density was $7.2{\;\times \;10}^{11}{\;\mathrm{cm}}^{-2}$, the electron effective mass was 0.02, and *λ_MFP_* was 500 nm. The black dots are the simulated current, *I*, for applied voltage, *V_0_*. The dashed line is what is predicted analytically from the Drude model for the given parameters.

To verify that the simulator accurately predicts current–voltage characteristics, it was run on a rectangular sample with no constrictions and then compared to the simple analytical solution from the Drude model [15]. Figure 2 shows the simulated and modeled current, *I*, for applied voltage *V_0_*. The mesh size was set to 250 nm and constant throughout the device, since the electric field was uniform due to the absence of constrictions. The simulated result agrees well with the analytical solution, which supports the accuracy of the simulation.

### 3.2. Inverse-Arrowhead and Z-Diode

**Figure 3:**
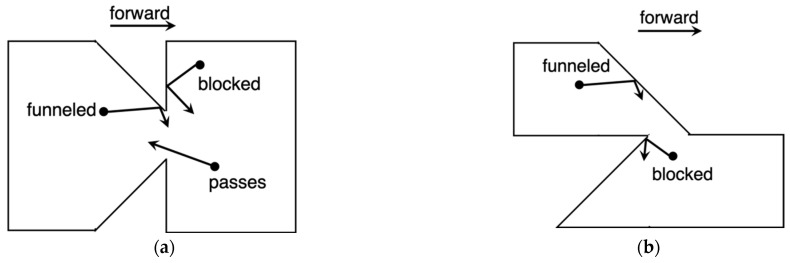
(**a**) Geometric effect in the inverse-arrowhead diode and (**b**) geometric effect in the Z-diode. In both cases, charges are funneled through in the forward direction and blocked in the reverse direction. The reverse blockage is more effective in the Z-diode.

After demonstration of the inverse-arrowhead diode, Zhu proposed that alternative geometries could present increased current asymmetry, with one in particular being the Z-diode [12], which is compared to the inverse-arrowhead in Figure 3. In both the inverse-arrowhead (Figure 3a) and the Z-diode (Figure 3b), under forward bias, charges drift from left to right, and the geometry is such that reflections off the device boundaries funnel the charges through the constriction, or neck. However, in reverse bias, there is a notable difference in the geometries of the two devices. As charges drift right to left, the inverse-arrowhead geometry allows for easier transmission through the neck because the opening is perpendicular to the general drift direction. This reverse leakage is reduced in the Z-diode because the neck opening is oriented perpendicular to the general drift direction.

**Figure 4:**
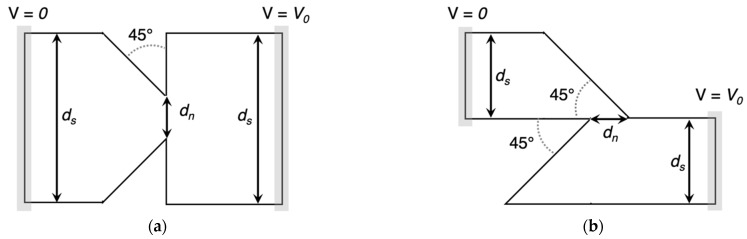
Relevant dimensions of (**a**) an inverse-arrowhead diode and (**b**) a Z-diode. The shoulder width is represented by *d_s_*, and the neck width is represented by *d_n_*. For electron carriers, the current, *I*, should be positive for a positive *V_0_*.

To compare the rectification capabilities of the two geometries, their *I(V)* characteristics were simulated. The relevant geometric variables are illustrated in Figure 4. The shoulders, i.e., the electrodes from which charges are injected, have width *d_s_*, while the neck, or the minimum constriction in the device, has width *d_n_*. The voltage, *V_0_*, is applied between the left and right electrodes to drive the current, *I*, through the device. The strength of the geometric rectification effect depends on the device dimensions relative to *λ_MFP_* [12]. Both simulations used *λ_MFP_* *=* 500 nm, *d_n_ =* 100 nm, and *d_s_ =* 1 μm. Voltages were swept from −0.5 to 0.5 V.

The material was assumed to be graphene for the simulation, such that the material properties can be tuned by applying a gate bias. Much graphene processing is carried out on thermally oxidized silicon wafers. In this case, the graphene device sits atop the oxide, and a gate voltage is applied to the silicon layer below the oxide. The dependence of carrier concentration on gate voltage can be described by $n=\epsilon_{0}\epsilon_{r}V_{g}/tq$ [16], where $\epsilon_{0}$ is the permittivity of free space, $\epsilon_{r}=3.9$ is the relative permittivity of SiO_2_, $V_{g}$ is the gate voltage relative to the Dirac point where the carrier concentration is minimum, *t* is the thickness of the SiO_2_ substrate, and *q* is the elementary charge. *V_g_* can be directly applied by biasing the substrate, but is often nonzero even without bias due to the unintentional impurity doping that occurs in fabrication [3,16,17]. In such cases, it is not uncommon for *V_g_* to be in the order of 10 V, so we set *V_g_* = 10 V for our simulations. The oxide thickness is set to *t* = 300 nm, since this is a common SiO_2_ thickness used in graphene processing. For these parameters, electrons are the majority carriers, and their concentration is $n=7.2\;\times \;10^{11}\;{\mathrm{cm}}^{-2}$. The effective mass, $m^{*}$, follows the relation,

(2) $$m^{*}=\sqrt{\frac{h^{2}n}{{4\pi m}_{0}^{2}v_{F}^{2}}}\;$$

where *h* is Planck’s constant, and $m_{0}$ is the electron mass [18]. For these simulations, this gives $m^{*}=0.02$. For all the simulations given here, we used a time step of *dt =* *τ*/10. The maximum mesh size was set to 100 nm at the shoulders and 40 nm at the neck. Reducing either the time step or the maximum mesh size further resulted in longer computation times for the same *I(V)* curves.

**Figure 5:**
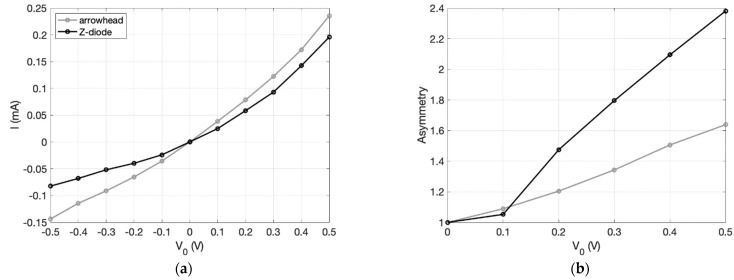
Comparison of inverse-arrowhead diode and Z-diode. (**a**) The current–voltage characteristics of both geometries and (**b**) the resulting current asymmetries. Both diodes have $\lambda_{MFP}=500\;\mathrm{nm}$, $d_{n}=100\;\mathrm{nm}$, and $d_{s}=1\;\mu m$. It is unclear why the asymmetry is slightly higher for the inverse arrowhead diode at 0.1 V. It is possible that it is an artifact of the simulator at low currents.

The resulting *I(V)* curves for both diodes, as well as their current asymmetries,

(3) $$Asymmetry=\left|\frac{{I(+V}_{0})}{I(─V_{0})}\right|$$

are shown in Figure 5. Both diodes show rectification behavior in the quasi-ballistic regime, where the charge carriers undergo momentum relaxation events. As expected, the Z-diode exhibits a more nonlinear I(V) curve and a higher current asymmetry above 0.1 V. This is predominantly due to the stronger suppression of the reverse current due the geometry being more effective at reflecting charge carriers away from the neck in reverse bias. Although it is not yet clear why, this Z-diode is less asymmetric at 0.1 V and below, indicating the existence of a turn-on voltage that must be supplied for substantial rectification. For the given dimensions, the Z-diode shows a higher resistance at low biases, which must be considered when impedance matching devices to other circuit components, such as antennas in rectenna circuits. In a recent study by Wang et. al, an inverse-arrowhead diode was fabricated and measured with results showing correspondence to the simulator [18].

### 3.3. Asymmetry Variation with λ_MFP_

**Figure 6:**
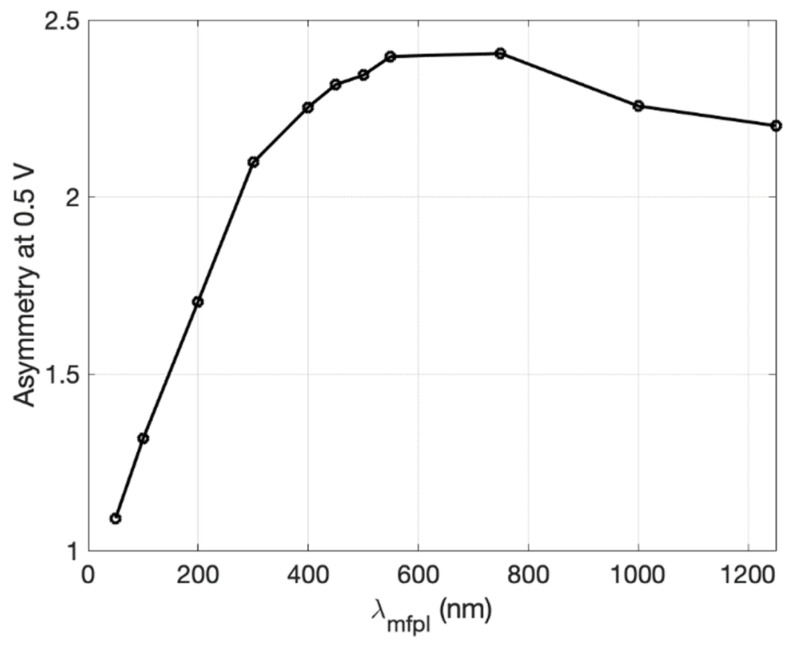
Asymmetry dependence on carrier mean-free-path length for a graphene Z-diode. For each *λ_MFP_*, the current asymmetry was sampled at 0.5 V. The diode dimensions are given by *d_n_* = 100 nm and *d_s_* = 1 μm.

In the fully ballistic regime, *λ_MFP_* is assumed to be larger than all device dimensions. In the quasi-ballistic regime, this is not necessarily the case, and so changing the device size or *λ_MFP_* changes the prevalence of momentum-destroying collisions during electron transport. To understand this effect, the previous Z-diode was simulated over a range of *λ_MFP_*. For each value of *λ_MFP_*, the currents were simulated for applied voltages of $V_{0}=\pm 0.5\;V$, and the resulting asymmetry was calculated, with the results shown in Figure 6.

As *λ_MFP_* approaches zero, the asymmetry approaches 1, which is what is expected for macroscopic conductors with large critical dimensions relative to *λ_MFP_* that do not exhibit quasi-ballistic transport. In such a case, the carrier momentum is destroyed too often for transport to be dominated by the device geometry. However, the most interesting revelation is that the current asymmetry peaks for a finite mean-free-path length, which has previously not been discussed in literature. In the case of the Z-diode with a 100 nm neck, the peak asymmetry appears to occur around $\lambda_{peak}\;\approx \;600\;\mathrm{nm}$, with asymmetry dropping for $\lambda_{MFP}{\;>\;\lambda }_{peak}$. This effect may be due to carriers with increasingly large *λ_MFP_* undergoing more reflections at device boundaries under reverse bias before having their momenta reset. A charge carrier that is initially deflected away from the neck can reflect off the bottom boundary and be directed toward the neck again by the strong electric field. If the carrier undergoes no momentum relaxation in the time frame, then its velocity will not be randomized before it travels through the neck and contributes to an increased reverse current. The value of *λ_peak_* is likely to change based on the applied voltage and the diode shape, and it may not exist at all for certain geometries.

## 4. Discussion

The simulation method described here presents several advantages compared to existing simulation methods. It is useful in studying the rectification behavior of diodes that operate in the quasi-ballistic regime and does not assume fully ballistic transport as do the other methods discussed in the introduction. In addition, it includes the effects of electric fields that result from inhomogeneous charge distributions and is computationally more efficient than existing Monte Carlo simulation methods.

It is important to note several factors and assumptions involved in the simulation that can compromise its validity in certain situations. This method assumes that the Fermi level of the charge carriers relative to the Dirac point, *E_F_*, is much larger than *k_B_T*. Here, *k_B_* is the Boltzmann constant and *T* is the ambient temperature. In this assumption, all of the carriers are assumed to have energy given by *E_F_* with negligible spread. In the case of graphene, the Fermi level is given by E_F_ = m^*^m_0_v_F_^2^ [19], which in our case gives *E_F_ =* 100 meV, which is larger than *k_B_T* by a factor of 4 at room temperature.

This simulation method also neglects quantum effects due to the wave nature of electrons. The de Broglie wavelength can be used to determine the length scales below which wave-like properties of the charge carriers become important. From the relation *E_F_ =* *ℏkv_F_* [19], where ℏ is the reduced Planck constant, and *k* is the carrier wave vector, we have *k* = 150 μm^−1^ for the above simulations, which translates to a de Broglie wavelength of 40 nm. This, along with the large coherence lengths reported for graphene [20], suggests that interference effects due to the wave nature of the electrons become important for device dimensions below the 100 nm scale. The de Broglie wavelength varies with the gate voltage, decreasing for greater *V_g_*, and can therefore be controlled to some extent.

At high electric fields, saturation effects due to high-energy charge carriers exciting optical phonons need to be accounted for [21]. For the parameters assumed in these simulations, i.e., *V_g_ =* 10 V for graphene on a 300 nm thick SiO_2_ substrate at room temperature, saturation effects become strong for applied fields around 0.5 V/μm [21]. For micron-sale devices, this simulation method should not be used with applied voltages exceeding the 1 V scale. The resulting current will be overstated as velocity saturation is ignored.

## 5. Conclusions

In our simulation approach, macroparticles are used to determine the potential and electric field at every mesh node in the device, and the particles move under the influence of the electrostatic forces. Each macroparticle’s momentum is periodically destroyed, and its direction is randomized before moving under the influence of the electric fields. The simulator is run over many collisions, and the current is derived from the rate at which the macroparticles traverse the device. By including momentum relaxation, it is applicable to quasi-ballistic transport as well as ballistic transport, and therefore, it can be used to predict the behavior of a wider range of devices than if the momentum relaxation was not included. It is also computationally more efficient than existing Monte Carlo simulation methods. The simulator has certain limits to its applicability. For very small device features, below 100 nm for the material properties used here, quantum effects can begin to take hold and cause erroneous simulation results. For graphene, the simulator will also overstate the current for electric fields over 0.5 V/μm due to its neglect of saturation effects.

Through simulation, we have studied a new geometric diode design, the Z-diode, which exhibits improved rectification over 0.1 V compared to the previous inverse-arrowhead design. It was found that the current asymmetry of such diodes is sensitive to the device dimensions relative to the carrier mean-free-path length. In particular, for a given Z-diode size, there exists a finite mean-free-path length that maximizes the current asymmetry at a particular voltage, which is a result that has previously not been discussed in the literature. Equivalently, this suggests that given a material with a specified mean-free-path length, the critical dimensions of the diode can be tuned to maximize asymmetry.

## Data Availability

The data presented in this study are openly available in FigShare at https://doi.org/10.6084/m9.figshare.16606217.v1 accessed on 23 July 2021.

## References

[1] Moddel G. (2008). Geometric Diode, Applications and Method. U.S. Patent.

[2] Moddel G., Zhu Z., Grover S., Joshi S. (2012). Ultrahigh speed graphene diode with reversible polarity. Solid State Commun..

[3] Zhu Z., Joshi S., Grover S., Moddel G. (2013). Graphene geometric diodes for terahertz rectennas. J. Phys. D: Appl. Phys..

[4] Sanchez A., Davis C.F., Liu K.C., Javan (1978). A. The MOM tunneling diode: Theoretical estimate of its performance at microwave and infrared frequencies. J. Appl. Phys..

[5] Grover S., Moddel G. (2011). Applicability of Metal/Insulator/Metal (MIM) Diodes to Solar Rectennas. IEEE J. Photovolt..

[6] Stearns J., Belkadi A., Moddel G. (2018).

[7] Banszerus L., Schmitz M., Engels S., Goldsche M., Watanabe K., Taniguchi T., Beschoten B., Stampfer C. (2016). Ballistic Transport Exceeding 28 μm in CVD Grown Graphene. Nano Lett..

[8] Singh A.K., Auton G., Hill E., Song A. (2015). Graphene based ballistic rectifiers. Carbon.

[9] Brownless J., Zhang J., Song A. (2020). Graphene ballistic rectifiers: Theory and geometry dependence. Carbon.

[10] Mladenovic D., Sandu T., Dragoman D. (2020). Electrical rectification in asymmetric graphene nanoribbons with pores. Physica E Low Dimens. Syst. Nanostruct..

[11] Berdiyorov G.R., Hamoudi H. (2021). Creating graphene geometry diodes through fluorination: First-principles studies. Comput. Mater. Sci..

[12] Zhu Z. (2014). Graphene Geometric Diodes for Optical Rectennas. PhD Thesis.

[13] Tskhakaya D., Fehske H., Schneider R., Weiße A. (2007). The particle-in-cell method. Computational Many-Particle Physics.

[14] The MathWorks, Inc. (2012). MathWorks MATLAB and Statistics Toolbox Release 2012.

[15] Ashcroft N.W., Mermin N.D. (1976). Solid State Physics.

[16] Novoselov K.S., Geim A.K., Morozov S.V., Jiang D., Zhang Y., Dubonos S.V., Grigorieva I.V., Firsov A.A. (2004). Electric Field Effect in Atomically Thin Carbon Films. Science.

[17] Tan Y.W., Zhang Y., Bolotin K., Zhao Y., Adam S., Hwang E.H., Das Sarma S., Stormer H.L., Kim P. (2007). Measurement of Scattering Rate and Minimum Conductivity in Graphene. Phys. Rev. Lett..

[18] Wang H., Jayaswal G., Deokar G., Stearns J., Costa P.M.F.J., Moddel G., Shamim A. (2021). CVD-Grown Monolayer Graphene-Based Geometric Diode for THz Rectennas. Nanomaterials.

[19] Novoselov K.S., Geim A.K., Morozov S.V., Jiang D., Katsnelson M.I., Grigorieva I.V., Dubonos S.V., Firsov A.A. (2005). Two-dimensional gas of massless Dirac fermions in graphene. Nature.

[20] Minke S., Bundesmann J., Weiss D., Eroms J. (2012). Phase coherent transport in graphene nanoribbons and graphene nanoribbon arrays. Phys. Rev. B Condens. Matter..

[21] Dorgan V.E., Myung-Ho B., Pop E. (2010). Mobility and saturation velocity in graphene on SiO_2_. Phys. Rev. Lett..

